# Transient transfection of insect Sf-9 cells in TubeSpin^®^ bioreactor 50 tubes

**DOI:** 10.1186/1753-6561-5-S8-P37

**Published:** 2011-11-22

**Authors:** Xiao Shen, Patrik O Michel, Qiuling Xie, David L Hacker, Florian M Wurm

**Affiliations:** 1 Laboratory of Cellular Biotechnology, Faculty of Life Sciences, Ecole Polytechnique Fédérale de Lausanne, CH-1015 Lausanne, Switzerland; 2 Institute of Bioengineering, Jinan University, Guangzhou 510632, Guangdong, P. R. China

## Background

Sf-9 cells, derived from *Spodoptera frugiperda*, are widely used for recombinant protein production using the baculovirus expression vector system (BEVS). However, this results in a productive viral infection and cell lysis. Therefore, a non-lytic, plasmid-based expression system for suspension Sf-9 cells would be a valuable alternative to the BEVS for rapid, scalable, and high-yielding recombinant protein production and for the generation of stable Sf-9 cell lines [4]. In this work, we present a simple, efficient and cost-effective plasmid-based method for transient expression of recombinant proteins in Sf-9 cells cultivated in serum-free suspension mode in a high-throughput culture system, TubeSpin^®^ bioreactor 50 tubes (TubeSpins).

## Materials and methods

Sf-9 cells were maintained in suspension in TubeSpins (TPP, Trasadingen, Switzerland) at 28 °C [5]. The human tumor necrosis factor receptor-Fc fusion protein (TNFR-Fc) gene was cloned into pIEx10 (Novagen, Merck, Darmstadt, Germany) to generate pIEx-TNFR-Fc. The cells in exponential growth phase were inoculated in fresh Sf900 II medium (Invitrogen, Carlsbad, CA) one day prior to transfection. The next day, the cells were transfected with 1.5 µg pTNFR-Fc and 2.25 µg linear 25 kDa polyethylenimine (PEI, Polysciences, Warrington, PA) per 10^6^ cells. The DNA and PEI were first mixed in de-ionized water at room temperature for 10 min prior to addition to the culture. At the time of transfection the cell density was 20 x 10^6^ cells per mL. Cells were subsequently diluted to 4 x 10^6^ cells per mL with fresh Sf900 II media to allow for growth. The culture was maintained at 28 °C in an incubator shaker for 7 d [5]. The TNFR-Fc concentration in the medium was determined by ELISA as described [6].

## Results

**Figure 1:**
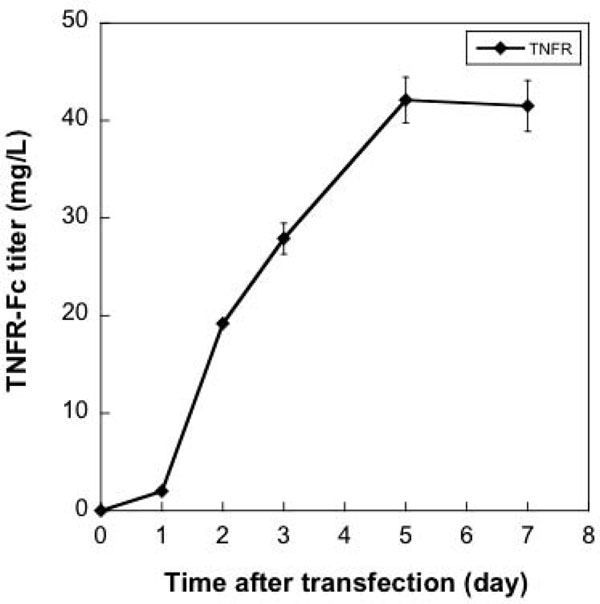
Sf-9 cells were transfected with pTNFR-Fc (■) plasmid DNA and PEI . The TNFR-Fc concentration in the medium was determined by ELISA at the times indicated.

Sf-9 cells were transfected in TubeSpins with pIEx-TNFR-Fc. By 7 d post-transfection, the TNFR-Fc concentration reached 42 mg/L (Figure 1). In a separate transfection with a plasmid expression the enhanced green fluorescent protein (GFP) gene, 58 % of the cells were GFP-positive at 5 d post-transfection (data not shown).

## Conclusions

This study validates the use of PEI for transient expression in suspension Sf-9 cell cultures. This system was used to produce both intracellular (GFP) and secreted (TNFR-Fc) proteins. The results show that PEI-mediated transient transfection is a fast and efficient alternative to BEVS for high-yielding protein expression in Sf-9 cells.
